# Implementing a cost effective and configurable hybrid simulation platform in healthcare education, using wearable and web-based technologies

**DOI:** 10.1186/s40561-022-00201-1

**Published:** 2022-05-20

**Authors:** Wayne J. Brown, Cindy Reid

**Affiliations:** 1 University of Eastern Finland, Joensuu, Finland; 2 Georgian College of Applied Arts and Technology, Barrie, Canada

**Keywords:** Wearable technologies, Hybrid simulation, Standardized patient, Healthcare education, Web-based technology

## Abstract

There are many examples of hybrid simulation models in healthcare education which are designed to simulate specific scenarios. However, there appears to be a need for a cost effective and configurable hybrid simulation platform which can be used by educators of various healthcare disciplines to simulate different scenarios. The purpose of this paper is to develop a proof-of-concept platform that can be easily implemented at little cost and provide flexibility to healthcare instructors to develop a variety of simulation scenarios, and to determine the effectiveness of this platform. Using a standardized patient, a person acting as a patient in a scripted manner, along with wearable and web-based technologies, a congestive heart failure simulation was used as an evaluative exercise for a group of personal support worker students at a Canadian Community College. Personal support workers typically provide care to any person who may require personal assistance with activities of daily living such as feeding, lifting, bathing, skin care and oral hygiene to name a few. Standardized patients are typically used in healthcare education to educate and evaluate soft skills, such as caregiver to patient communication, professionalism, as well as hard skills, such as history taking, examination and diagnostic skills (Rosen in J Crit Care 23:157–166, 2008). Instructor feedback indicated that the platform was easy to use and capable of simulating a large variety of scenarios. Pre and post test results are evidence of initial findings of promise indicating that the platform seemed to be effective in enabling students to meet learning outcomes. Focus group results seem to indicate an increase in student confidence as it relates to their ability to handle a similar scenario in the workplace.

## Introduction

Hybrid simulation models for healthcare education have been predominantly designed to simulate specific medical scenarios or procedures, such as tracheotomy insertion and cleaning (Dunbar-Reid et al., 2015; Verma et al., 2011), safe patient transfer (Lebel et al., 2018) and intravenous insertion (Devenny et al., 2018) to name a few. Many of these focused hybrid simulations employ either wearable technologies, standardized patients, web-based technologies, or some combination of all three. Unver et al. (2018) defines hybrid simulation as the “combination of more than one simulation modality in a single teaching or evaluation exercise”. Most hybrid simulation models discussed in the literature are limited, by design, in their scope, in that they are designed for a specific scenario. High fidelity simulators, on the other hand, such as SimMan 3G, bundled with the support software and optional packages, can be quite versatile yet can be cost prohibitive for many institutions, coming in at approximately 70,000 USD per device (Alsaad et al., 2017). Within the healthcare education context, Tuzer et al. (2016) defines simulation as “a realistic teaching/learning strategy that could be used to promote cooperation and communication between the disciplines without any risk of harm to patients” as compared to training with mannequins alone, which provides minimal opportunity to practice interpersonal communications (Devenny et al., 2018). There is much evidence in the literature for a need to further increase the realism, or fidelity, of simulation scenarios (Dunbar-Reid et al., 2015). Indeed, Dunbar-Reid et al. (2015) posits that providing tools for the healthcare instructors to enhance simulation fidelity ‘will, in overcoming the criticism of limited realism in simulation, allow educators to teach and assess interpersonal competency during simulation exercises’. Dunbar-Reid et al. (2015) goes on to say, ‘when it comes to simulation training, it is important that the simulation mirrors real life scenarios as close as possible, such that the actions and words of healthcare students approximate what is expected in actual clinical situations. Simulation in general has been shown to provide a realistic learning environment that can be controlled by the educator (Unver et al., 2018), thus minimizing environmental distractions and allowing for uninterrupted feedback (Tuzer et al., 2016). Finally, hybrid simulation has been shown to promote higher skill performance, knowledge acquisition, expansion of critical thinking and self-confidence (Unver et al., 2018).

During research conducted at Acibadem University, Istanbul, Turkey, hybrid simulation participants reported that the simulation’made it possible to develop a connection between their education and real-world situations’ while at the same time feeling more motivated towards their studies (Unver et al., 2018). Indeed, simulations are a recommended approach to healthcare education, in integrating communication and psychomotor skills, as these skills are intertwined in a clinical environment (Basak et al., 2019). Additionally, simulations have been integrated in recent times into nursing education programs for the purposes of improving student’s self-confidence in clinical practice (Unver et al., 2018).

This research focuses on the effective use of hybrid simulations in healthcare education, with the following overall research question: How can healthcare education, through simulation, be enhanced using wearable and web-based technologies and standardized patients? This research explores whether a low cost, configurable hybrid simulation platform (a set-up which combines wearable and web-based technologies with a standardized patient in a flexible format to support multiple healthcare simulations) can be built using readily available web-based and wearable technologies, and whether such a platform can be used in multiple healthcare education simulations for a variety of healthcare disciplines. As this is a hybrid simulation platform, the use of a standardized patient is an integral element to the effectiveness of this approach. The literature supports the idea that the use of standardized patients is a beneficial way to teach high-level knowledge and skills (Tuzer et al., 2016). The use of standardized patients in nursing education, for example, has become very popular in recent years (Basak et al., 2019). Additionally, research has shown that the use of a wearable device with a standardized patient allows the patient to appropriately respond to student’s questions as well as react appropriately within the context of the scenario (Devenny et al., 2018).

This paper first explores the general background of hybrid simulations and standardized patients as well as describes the research methodology and context in which the data for this research was gathered, followed by the results of the research. A discussion is then presented on how others can benefit from this research as well as the boundaries of the research relative to the inferences drawn from the paper, finally recommendations for further research are suggested.

### General background of hybrid simulation and standardized patients

One form of hybrid simulation incorporates a standardized patient with a supporting piece of simulation equipment such as a desktop model of a body part or section. This approach is often used to present to the learner a scenario in which the realism or fidelity of the set-up is, as much as possible, in compliance with real life (Basak et al., 2019).

The use of hybrid simulations in healthcare education has been present for several years to train a variety of medical personnel, such as paramedics, nurses, and doctors, to name a few (Lebel et al., 2018, Kutzin et al., 2017; Unver et al., 2018). Unver et al. (2018) found that students participating in a hybrid simulation training session better recognized the significance of communication and cooperation with team members. The same participants reported that the use of hybrid simulations improved their self-confidence, critical thinking, and decision-making skills. Unver et al. (2018) also found that their hybrid simulation was so realistic that students reported a feeling that they were ‘real nurses’ during the simulation. Indeed Dunbar-Reid et al. (2015) argues that more authentic learning opportunities are made available to students with the use of hybrid simulations due to the ‘enhanced physical, functional and psychological fidelity’. Tunzer et al. (2016) concluded that hybrid simulation as a teaching modality should be ‘integrated into nursing curriculum as an active learning methodology’ that is linked to clinical practice. Unver et al. (2018) found that integrating hybrid simulation experiences throughout a nursing curriculum was seen as a positive experience by students. Similarly, Nassif et al. (2017) found that the use of hybrid simulation fosters a perception of ease and comfort by the students which could boost the student’s confidence in their ability to effectively communicate with the patient. Indeed, hybrid simulation was a recommended approach to healthcare education by the participants of the Hybrid Simulation in Teaching Clinical Breast Examination study over the use of a tabletop model (Nassif et al., 2017).

As stated earlier, standardized patients are an integral aspect of a hybrid simulation scenario, adding to the overall fidelity of the simulation. Prior to the concept of hybrid simulation, standardized patients, sometimes called human actors, were used alone to create education simulation scenarios, an approach that dates as far back as 1963 (Rosen, 2008). In the 1970s, ‘patient actors’ were covertly integrated into the clinical setting to help validate the use of actors in healthcare education (Rosen, 2008). Around the same time, Paula Stillman of the University of Arizona was using simulated mothers in pediatric clerkship. This approach morphed into the use of actors with ‘chronic stable findings’, actors that Stillman called patient instructors (Rosen, 2008). This use of simulated patients into testing situations ushered in the current term ‘standardized patient’ (Rosen, 2008). In 1990/91 the Educational Commission of Foreign Medical Graduates used standardized patients in a pilot test for Foreign Medical Graduate certification exams (Rosen, 2008). Since that time, standardized patients have proven to be an important part of healthcare education as they add significant realism to any simulation.

The use of standardized patients has been shown to be effective in increasing the knowledge level of students in examination techniques (Tuzer et al., 2016). Additionally, the literature supports the notion that the use of standardized patients in simulations may help students communicate better with real patients during stressful clinical scenarios (Ignacio et al., 2015).

Basak found that simulations conducted using standardized patients provided the students with a realistic learning opportunity which is in keeping with the goals of healthcare education; to ensure that students are prepared for the real world (Basak et al., 2019). Similarly, Cowperthwait et al. (2015) found, through observation, that students interacted more readily with standardized patients as evidenced by their willingness to ask questions, offer reassurance, and explain procedures as compared to the same students working exclusively with mannequins. Coffey et al. (2016) found that standardized patients offered a more realistic experience for learners as compared to mannequins and theorized that this level of realism would promote patient-caregiver interactions that would closely approximate real clinical practice. Coffey et al.(2016) 2015) also found that the use of standardized patients encouraged more humanistic interactions, such as touch.

Traditionally, mannequins have been used to represent acutely unwell patients for training and assessment of technical skills, however, there appears to be a shift towards the use of standardized patients as an innovative teaching method that has rapidly become widespread in recent years (Basak et al., 2019). Hybrid simulation with standardized patients has been used to develop specific simulation scenarios, however there was no evidence found in the literature that a generic hybrid simulation approach has been developed that could be used in multiple simulation scenarios.

### Enhancing healthcare education

From the previous discussion it can be seen that hybrid simulations using standardized patients have been effective yet very focused on specific training scenarios. This focus, and hence lack of generalization, led to the research question “How can healthcare education, through simulation, be enhanced using wearable and web-based technologies and standardized patients?” By ‘enhanced’ we mean, how can the hybrid simulation approach be repurposed so that it is easily configured by healthcare instructors, is affordable, and is flexible enough to be used by a variety of healthcare disciplines.

To better understand the effectiveness of a configurable hybrid simulation approach and, more importantly, to measure that effectiveness, two more specific research questions are articulated to gauge the overall effectiveness of this configurable approach:

1. What effect does a wearable and web-based hybrid simulation platform have on the ability to equip students to meet selected course learning outcomes?
2. What effect does a wearable and web-based hybrid simulation platform have on improving the confidence of students in their ability to handle real life medical scenarios?

To answer these questions, a simple and affordable hybrid simulation platform was developed in this research, based upon wearable and web-based technologies. The technologies enabled for a configurable platform to be used by healthcare instructors in a variety of healthcare disciplines. The effectiveness of the platform was tested through a simulation experimental study, where personal support worker students at a Canadian college, selected through a convenience sample, participated in the simulation experiment. A pre and a post test, as well as a focus group, were employed to determine if the students were able to meet selected course learning outcomes and ascertain improvement in the student’s view of their own confidence because of the simulation approach. The selected course learning outcomes relative to this research were as follows:

1. State the signs of congestive heart failure.
2. Identify congestive heart failure (CHF) as a possible cause of patient unwellness by asking appropriate diagnostic questions.
3. List the symptoms of congestive heart failure.
4. Ensure patient comfort upon discovery of congestive heart failure?

To capture the appropriate data, a research design was developed based upon a case study approach using mixed methodologies. Qualitative data was captured using a focus group, while quantitative data was captured using pre and post tests.

### Research methodology

The simulation experimental study took place at a community college located in central Canada. The experiment included nine phases as outlined in Table 1:

**Table 1:** Research Phases

Phase	Duration
Student cohort identified	2 weeks
Ethics approval secured	4 months
Research information provided to student group	1 week
Consent secured from the student group	2 weeks
Pre-Test Administered	30 min
Hybrid Simulation Experiment Conducted	1 h
Post-test administered	30 min
Focus group completed	1 h
Data analyzed	1 month

### General description of the experiment

This study was based upon a limited convenience sample of six personal support worker students, selected from a class of 10 students, enrolled in a one-year college certificate. The approximate age of the sample ranged from nineteen years to twenty-four years of age. Of the six students who participated in the experiment, five were female and one was male. The highest academic credential attained by all six students upon entering the program was high school.

This quasi-experimental approach utilized a one-group pre-test/post-test methodology. Data were collected during the month of April of 2021; data collection took place during the Covid-19 pandemic. Government regulations at the time of data collection allowed only ten individuals to be together in person indoors within an educational institution, and then only wearing the necessary personal protective equipment. To maintain the ten-person limit, seven students were selected for the study, which along with the instructor, standardized patient and principal investigator kept the total count to ten. Just prior to the commencement of the study, the seventh student elected not to participate, bringing the total count to nine.

Prior to the administrative work of the study, i.e., recruitment and associated activities, ethics approval was secured from the college research ethics board. Student participants were provided with the research overview and necessary consent forms. It was decided by the course instructor and the principal investigator that congestive heart failure would be an appropriate scenario to present to the students, as this is a very common scenario that the students would likely encounter in the workplace. A local professional actor was hired to play the role of the standardized patient.

Prior to the simulation, the actor was given a briefing on congestive heart failure and was also provided with three cues to rehearse. Each cue was associated with how a typical person in congestive heart failure presents:

- Cue 1: spontaneously sitting up and finding it hard to breathe.
- Cue 2: spontaneously sitting up and needing to go to the washroom.
- Cue 3: sudden onset of severe shortness of breath.

The actor rehearsed each cue prior to the simulation to ensure accuracy and realism.

**Fig. 1:**
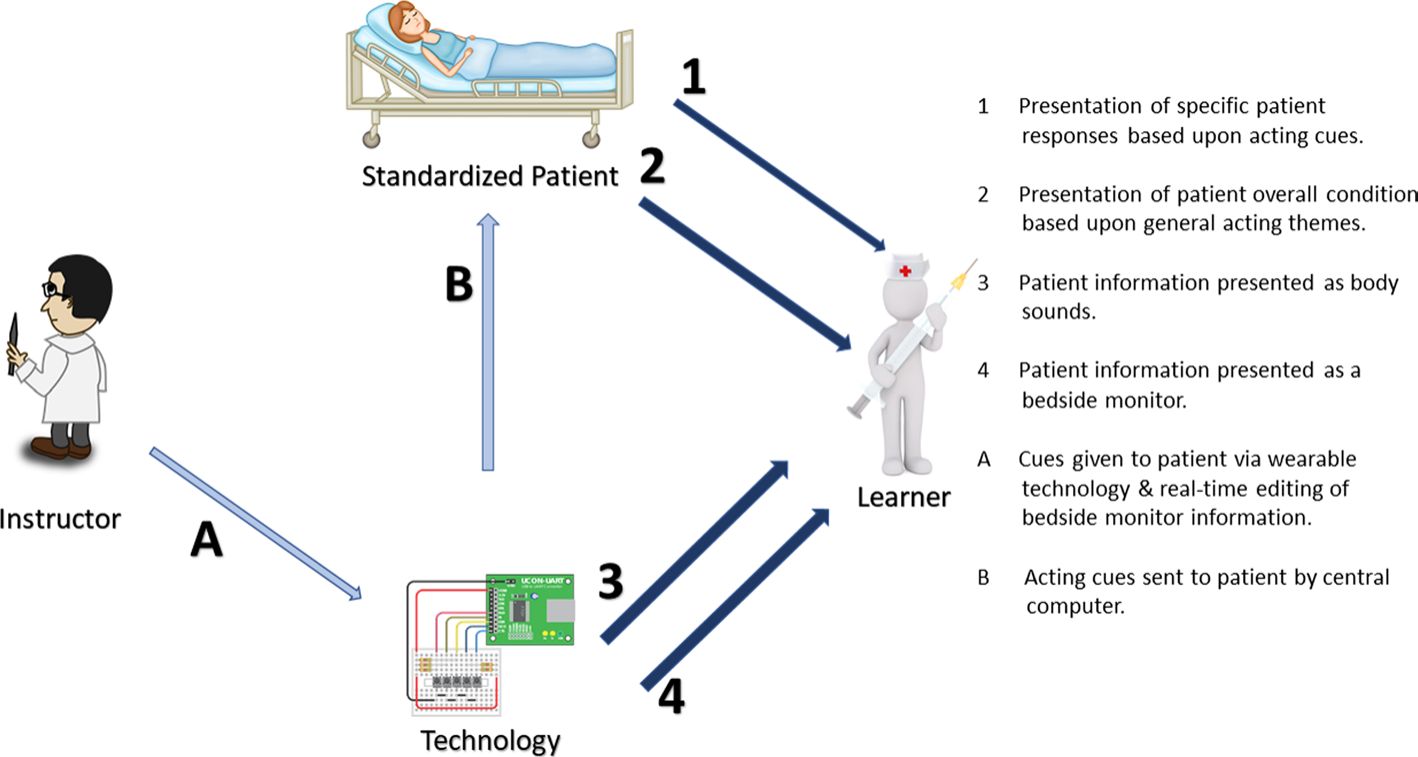
Hybrid simulation theoretical framework

Figure 1 (Brown and Kinshuk, 2022) illustrates the theoretical framework that the simulation platform was based upon. This approach allows the student to receive relevant patient information from four different channels of communication (marked with numbers and red arrows in Fig. 1). This framework was developed to provide a configurable hybrid simulation approach using a standardized patient in conjunction with wearable and web-based technologies.

In the simulation platform, channel one provided information to the students in the form of patient responses based upon specific acting cues provided to the standardized patient by a member of the research team. During the simulation, one of three cues were given to the actor by the principal investigator via an Arduino MKR1010 microcontroller. Readily available Arduino software from the WiFiNINA library ((c) 2020 K. Söderby for Arduino) was used to control the vibration motor through a wi-fi connection. All electronic connections were completed by the research team in consultation with standard Arduino documentation.

**Fig. 2:**
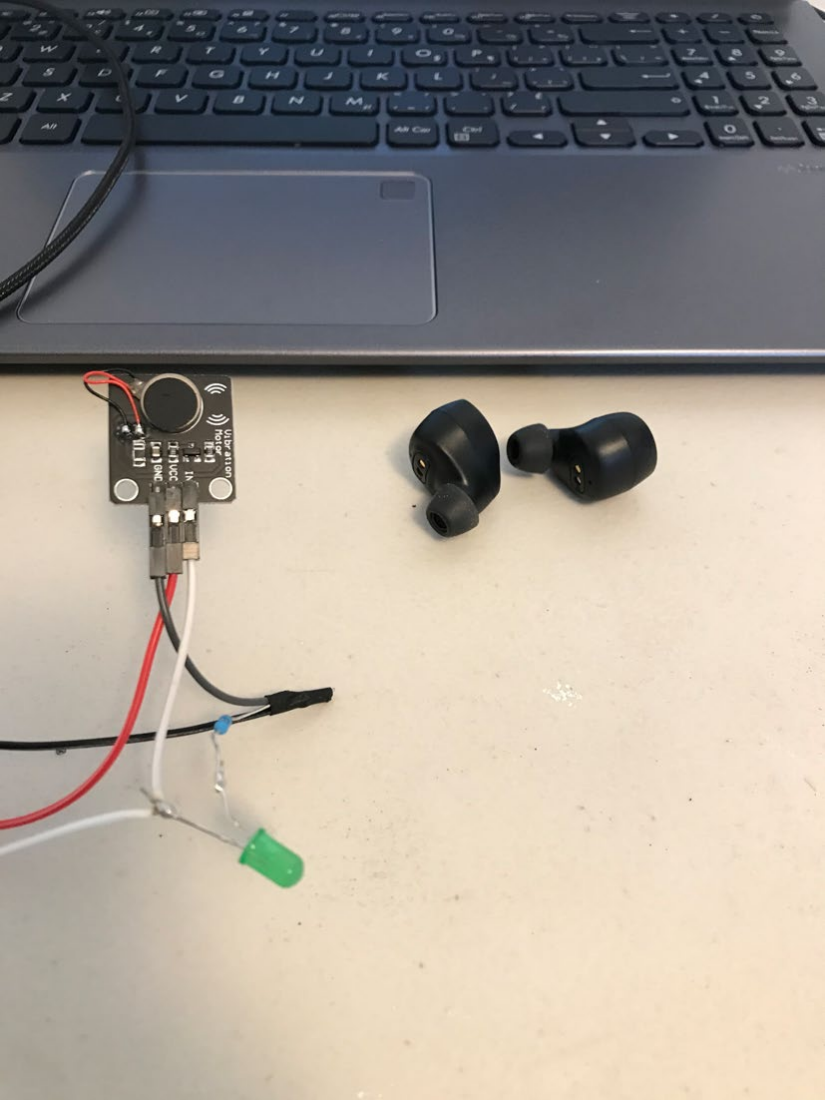
Vibration motor used to cue the standardized patient and Blue-tooth earbuds used to present an elevated heartrate

**Fig. 3:**
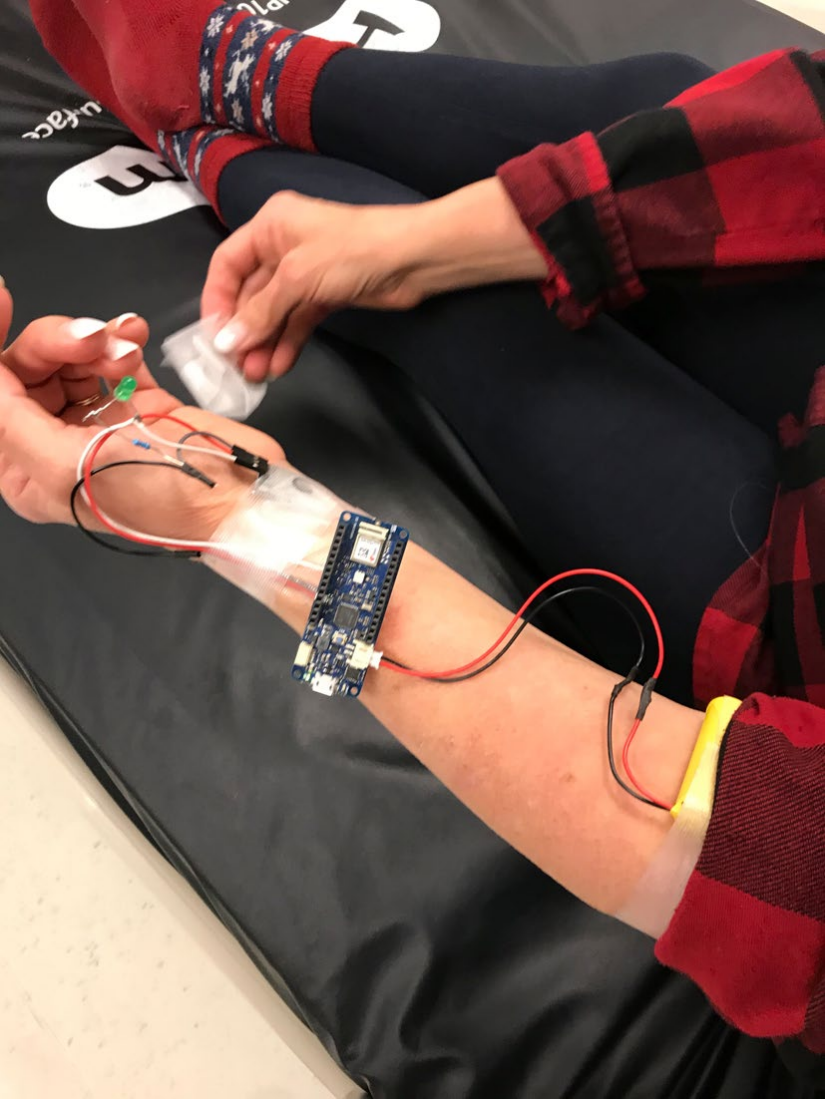
Vibration motor, Arduino and battery pack ‘attached’ to the standardized patient

**Fig. 4:**
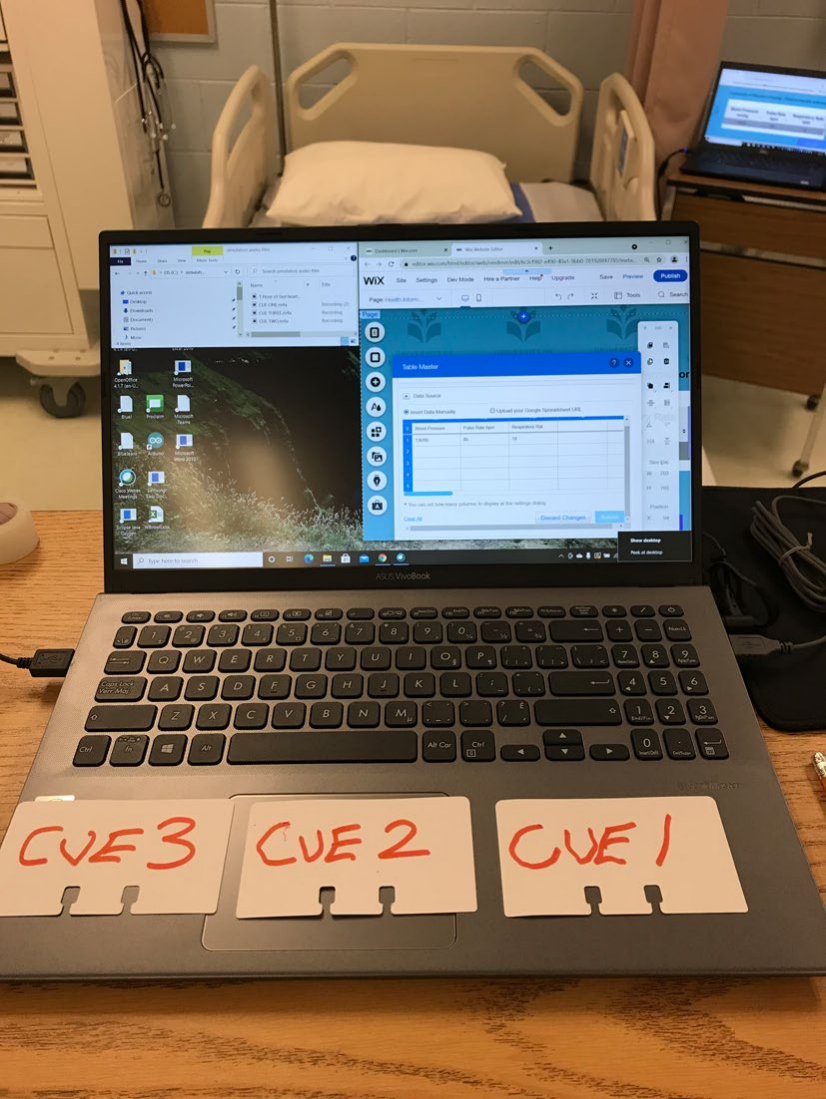
Simulation control computer showing WIX editor, audio files and manual cue cards

When the microcontroller was remotely triggered, it activated a vibration motor which was taped to the standardized patient’s arm. The vibration was felt by the standardized patient, causing her to act accordingly. The number of vibrations was mapped to the appropriate cue, in that one vibration meant cue one, etc. As a back-up, simple cue cards were also created by the research team and made available to the principal investigator, so that they could be held up in line of sight of the actor in the event of a technology failure. The usefulness of this back up system became evident when the Arduino MKR1010 microcontroller lost Wi-Fi connection part way through the simulation! (Figs. 2, 3, 4).

Channel two provided information to the students in the form of overall patient presentation based upon the overall acting theme provided to the actor prior to the simulation, which aligned with congestive heart failure. This included information such as a history of family heart issues, general fatigue and difficulty breathing. Again, the actor rehearsed this information prior to the simulation to ensure maximum realism.

The third channel provided information to the students that was only discernable with the use of a stethoscope. In keeping with the theme of congestive heart failure, an audio file of an elevated heartbeat was secured from You-Tube and played through a Blue-tooth ear bud that was taped to the patient’s skin over the heart and under the clothes. When the student attempted to listen to the patient’s heart, the audio file was played, presenting the student with an elevated heart rate.

The fourth and final channel of communication to the student took the form of a bedside monitor. In the world of healthcare, this is typically a computer screen that is mounted on a pole or a moveable equipment cabinet, and displays vital patient information such as heart rate, blood pressure and respiration rate. This channel of communication provided maximum flexibility and configurability in that any relevant information that was in keeping with the simulation scenario could be displayed and edited in real time. The bedside monitor display was designed using the free features of a website development software called WIX. This software allowed the researchers to develop a simple display showing applicable patient information. Once the site was developed, it was ‘published’ to the internet which allowed the site to be viewed using a browser on a second laptop connected to the internet, this second laptop acted as the bedside monitor display. The data was changed and updated in real time using the online editor built into the WIX website development platform.

**Fig. 5:**
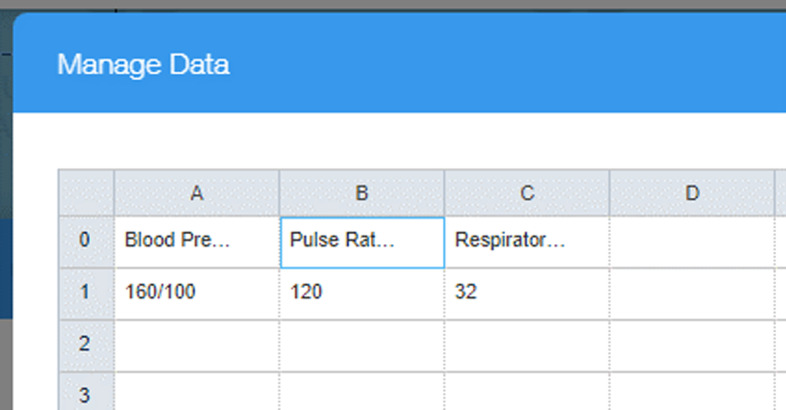
nWIX data editor

**Fig. 6:**
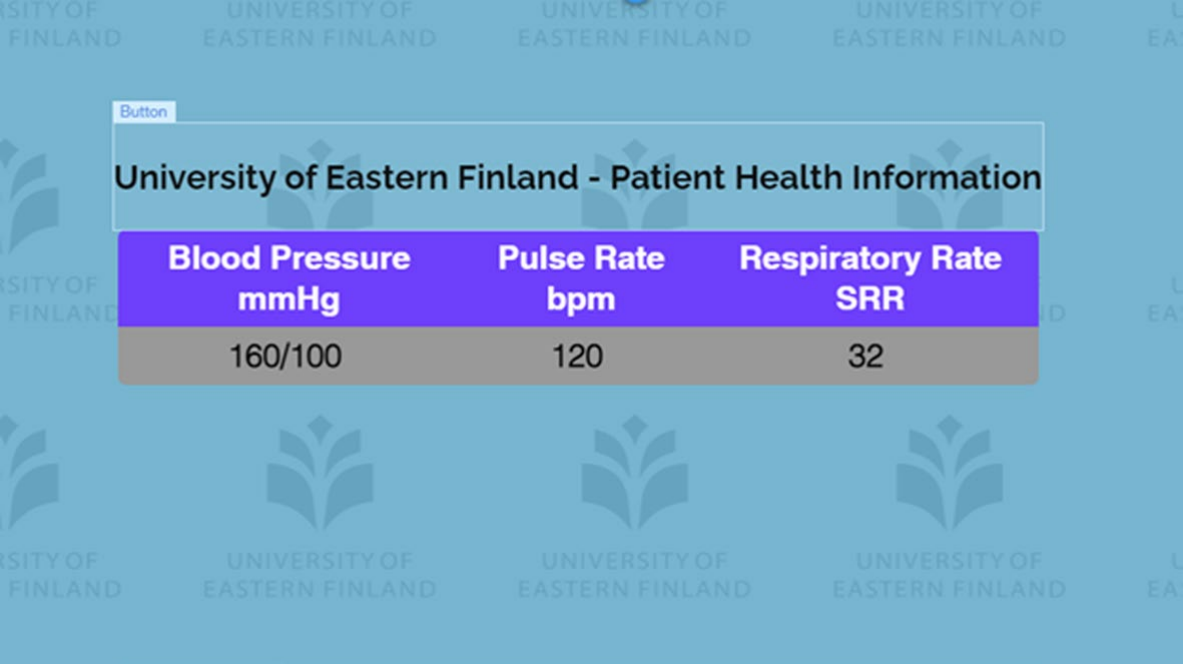
Patient health information displayed on the bedside monitor

**Fig. 7:**
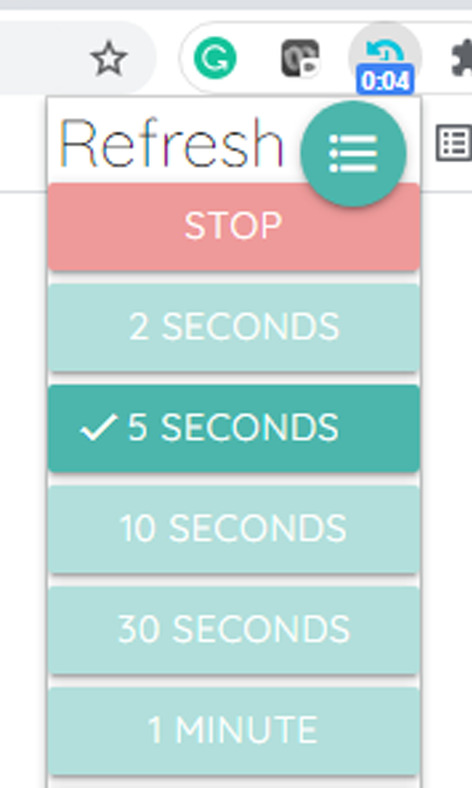
Google chrome auto-refresh plug-in

One of the challenges encountered during the initial design of the simulation was that changes made to the data did not show up on the bedside monitor unless the website in which the data was displayed was manually refreshed. This obstacle was overcome by simply installing an auto refresh plug-in on the Google chrome browser. This plug-in allowed the researchers to set the refresh time in the range of two seconds to sixty minutes, for this research a ten second interval was chosen as an appropriate refresh rate. This allowed enough time for the principal investigator to make changes to the data and publish the new data between refresh cycles, as required (Figs. 5, 6, 7).

Fifteen minutes prior to the simulation, the participating students assembled in an adjoining classroom to complete the pre-test. Students were provided with pen and paper and given approximately ten minutes to complete the pre-test. However, most students completed the pre-test in just a few minutes as they were not able to answer, in any detail, most of the questions on the pre-test.

Once the pre-test was completed, students moved to the simulation room where the standardized patient (the actor) was already laying on the hospital bed. The instructor conducted a short briefing prior to the commencement of the simulation, providing the students with the context of the simulation (a client in the community where home care is required) and the applicable patient information. Because the students were early in their program, the instructor elected to use the scenario as an ‘instructor facilitated’ simulation, where the instructor coached the students as a single group through the scenario, prompting them regarding what to ask the patient, and what to look for and observe. This approach is indeed different from that of a ‘student-experienced’ simulation where the student(s) are left alone to navigate the simulation in its entirety, thus determining themselves what questions to ask and what course of action to take as they observe and gather the necessary patient information. At the same time, the principal investigator was changing patient information as displayed on the bedside monitor and cuing the standardized patient with one of the three cues as previously rehearsed. The evidence of the actor’s authenticity bore out when she received cue two, spontaneously sitting up and needing to go to the washroom. Both the instructor and the students thought that the actor really had to go to the washroom and thus were prepared to take a break from the simulation! Ironically, the instructor thought the need to use the washroom was not part of the simulation even though it was an agreed upon cue and discussed prior to the commencement of the simulation! This indeed speaks to the acting skills of the standardized patient, the realism of the simulation and the instructor’s immersion in the moment as a professional healthcare provider. Indeed, it speaks to the advantage of employing a standardized patient. A similar effect was evidenced by Dunbar-Reid et al. (2015) where there appeared to be a blurring of the boundaries between simulation and reality.

Upon completion of the simulation, students were again assembled in the adjoining classroom to complete the paper and pen post-test. The same four questions were asked of the students on the post-test that were asked during pre-test. However, all students took the full allocated ten minutes to complete the test providing more detailed answers to each question.

**Fig. 8:**
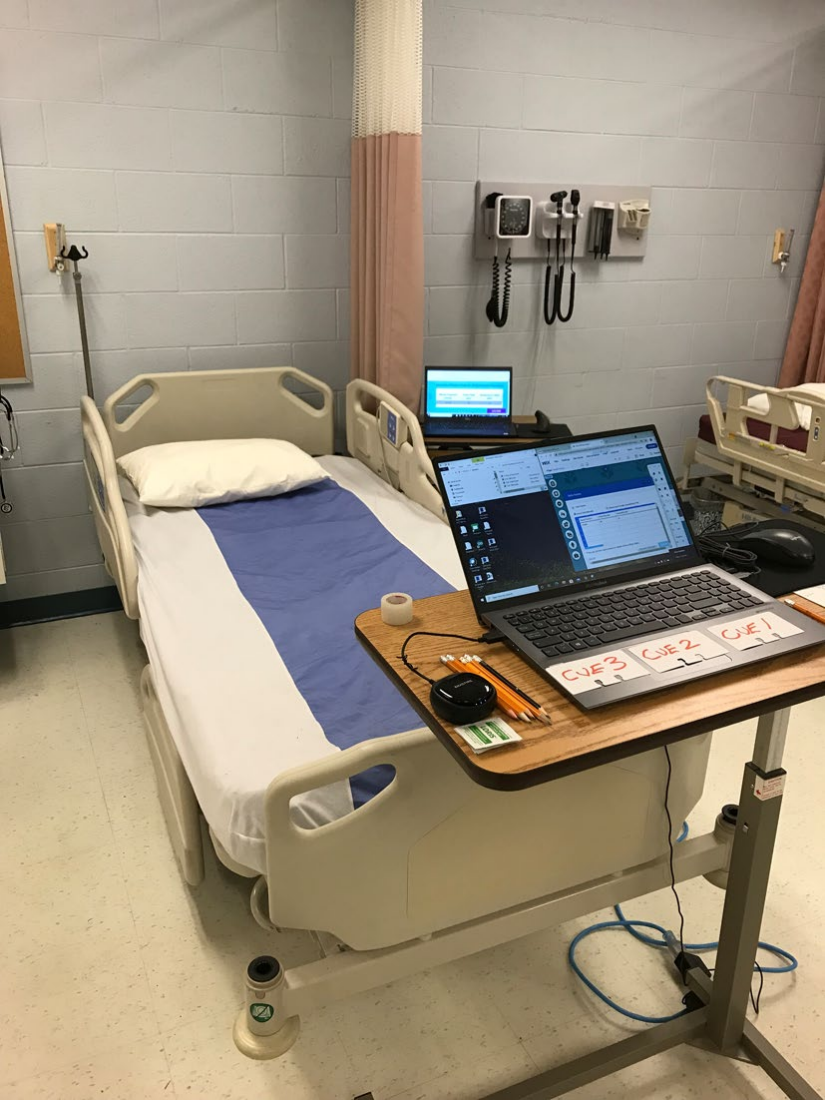
College simulation set-up

The following evening a virtual focus group was held using the Webex online meeting system. The focus group was held virtually due to Covid-19 protocols. Unfortunately, only three students participated in the focus group, which lasted for approximately thirty minutes. During the focus group the participants were asked the following four questions:

1. What are your initial thoughts on today’s simulation experience?
2. What are your thoughts on the realism of the simulation, and what aspects of the simulation do you feel contributed to that realism?
3. Do you feel that you will have more confidence at work, if presented with a scenario similar to that of the simulation? If so, why do you feel that you will be more confident?
4. Do you have any closing thoughts or remarks for the researchers? (Fig. 8)

## Results

In the pre-test, three of the six students had submitted a blank test indicating no knowledge of congestive heart failure relative to the signs of congestive heart failure and the symptoms of congestive heart failure. Of the three other pre-tests completed, one demonstrated minimum knowledge and the other two showed a moderate amount of knowledge.

Pre-Test Questions (Table 2):

1. State the signs of congestive heart failure.
2. What question(s) can be asked of a patient’s wellness to possibly identify congestive heart failure as the root cause?
3. List the symptoms of congestive heart failure.
4. What techniques can be employed to ensure patient comfort upon discovery of congestive heart failure?

**Table 2:** Pre and post-test results

	Question 1	Question 2	Question 3	Question 4
*Pre-test*				
# of students answering correctly	3	3	1	1
*Post-test*				
# of students answering correctly	5	6	6	2

To address the first research question of this study, “What effect does a wearable and web-based hybrid simulation platform have on the ability to equip students to meet learning outcomes?”, the analysis of the pre and the post-tests results indicate that the simulation appeared to enable students to meet learning outcomes, in that students were able to provide more correct answers on the post- test than pre-test. On the pre-test, three of the students answered question one correctly; stating the signs of congestive heart failure whereas in the post test, 5 of the six students answered correctly. The results are almost the same for the second question regarding the patient’s wellness identification; on the pre-test, three of the students answered the question correctly, whereas in the post-test, all six students answered the question correctly. In the pre-test only one student was able to provide the correct symptoms of congestive heart failure whereas in the post-test al 6 students were able to answer correctly. And finally, on the question of techniques which can be employed to ensure patient comfort upon the discovery of congestive heart failure, only one student was able to answer this question correctly, whereas in the post test, 2 students gave the correct answer. It should be noted that ethics approval did not allow the collection of any personally identifiable information from the pre or post-test, and therefore the data could only be compared as a group versus as individual students. These test results give strong indication that the configurable hybrid simulation platform used as an intervention enables students to meet course learning outcomes. These results address the first research question.

To address the second research question, “What effect does a wearable and web-based hybrid simulation platform have on improving the confidence of students in their ability to handle real life medical scenarios?”, the data from the virtual focus group was analyzed based upon an epistemological orientation where themes were identified in the transcript that emphasized where group members tended to collaborate on the same shared meaning or perspective (Stewart et al., 2007). All three students, who took part in the focus group, reported that the simulation was very realistic. As one student reported “it felt very real when the patient sat up with shortness of breath”. Students reported the ease with which they were able to communicate with the patient because the patient was able to talk back. One student noted that the “ability to talk with the patient vs a mannequin eliminated the awkwardness from the simulation”. This ease of communication may improve the student’s future ability to ‘connect’ with their patients, as a patient’s confidence in their caregiver has been shown to be greatly influenced by the caregiver’s ability to interact in an effective and re-assuring way with the patient (Devenny et al., 2018).

As previously described, the use of a standardized patient, is worth reflecting on once again as evidence of the realism of the scenario for both the students and the instructor. Students noted that they found it difficult to discern between simulation activity and real-life activity. One student commented that “the actor was very good at her job, making the simulation very real for me.” Students reported that this simulation enabled them or made them feel more confident in their abilities to handle congestive heart failure in the real world, noting that the simulation felt real at times and thus “removed some of the fear of facing a similar situation in the community.” Two students noted how the bedside monitor added to the realism of the simulation. These comments give a strong indication that the students gained confidence because of the simulation, and they felt that this confidence will be brought into the workforce. Feedback from focus group participants indicated that all students perceived that the use of a standardized patient along with the supporting technologies provided a very realistic learning experience that closely approximated a real-life scenario. Students found that the use of a standardized patient enabled them to communicate with the patient more freely and indeed carry on a conversation with the patient as it related to the scenario.

## Conclusion

Reflecting on the research questions, one can see from the previous discussion that hybrid simulation platforms have the potential to enhance healthcare education by providing a cost effective and flexible solution. Specifically, the pre and post test results give hopeful indication that the platform may be effective in equipping students to meet course learning outcomes. The focus group results indicate that hybrid simulations can be effective in improving the perceived confidence of students in their ability to handle real-life medical scenarios.

Pre and post-test results provide initial findings of promise, that this simulation platform enabled students to meet course learning outcomes. Both the pre and post-test were completed by all six students. These results suggest that the configurable hybrid simulation platform may be an effective tool to enable students to meet learning outcomes and to build confidence in their ability to handle similar real-world scenarios. After participating in the simulation, students were able to answer post-test questions with the rigour that aligned with accepted knowledge of the subject or were able to provide more detail in their answers as compared to answers provided in the pre-test. During the focus group students expressed an improved confidence in their abilities to handle similar scenarios in the field upon graduation.

The total cost of the hybrid simulation platform (excluding the cost of ubiquitous items such as laptops and tablets) was approximately $300. This cost was incurred in the purchase of an Arduino microcontroller and associated peripherals used to cue the patient along with quality Bluetooth earbuds to provide stethoscope discernible sounds relative to the scenario. The web page authoring tool, WIX, did not incur any cost since only basic functions were required to develop a bedside monitor which were available in the free version of the tool. This research has shown that off the shelf hardware and software can be packaged into a hybrid simulation platform that can serve as a flexible, configurable teaching tool that can be easily employed in multiple disciplines for multiple scenarios. Considering its capabilities and cost, this simple yet flexible and easy to use hybrid simulation platform may be an appealing alternative for resource constrained educational institutions.

The findings of this study are strong indicators that this type of simulation platform may be effective in helping students meet course learning outcomes and build confidence in their ability to handle similar scenarios in a clinical situation across multiple healthcare disciplines.

This research also indicates that the use of a flexible hybrid simulation platform based upon wearable and web-based technologies can be easily employed by the instructors and is very affordable for the institutions. This research employed a hybrid simulation platform to simulate a patient presenting with congestive heart failure to first term personal support worker students. The ease of use and affordability of the platform makes it a compelling tool to employ in any health-based education curriculum to enable students to meet a selection of course learning outcomes. The adaptability of the platform to a variety of scenarios within different health-care disciplines may make this a compelling approach for a diversified cross-section of learning institutions.

The findings of this study are limited, as the research was conducted with only six students from one healthcare discipline, based upon one simulation scenario. Additionally, of the six students, only three were able to participate in the focus group which restricts the generalization of the conclusions regarding student’s confidence and would require further studies with larger groups of students. A convenience sample was used to test the simulation platform as this was the only group of students available during the simulation timeframe which was during the world-wide Covid-19 pandemic. As this simulation was tested with first term students the results may be different for other disciplines or more advanced students.

While this research has provided valuable insights into the effectiveness of hybrid simulations in educational settings, the context, and the restrictions in which this research was conducted have led to several future research recommendations. The first of these includes the testing of this simulation approach with a larger group of students to ascertain a greater cross-section of student type and abilities. Additionally, this research was conducted with first semester students; it would be helpful to conduct similar research with more advanced students to measure the attainment of learning outcomes and building of confidence. Research could also be conducted around the building of the student’s confidence by interviewing the students several months after graduation to get a sense for the students’ confidence in the workplace and how that confidence can be tied back to the simulation experience. Furthermore, expansion of the platform could be developed to determine the limits of the platform in providing more complex scenarios with multiple patient cues, more bedside monitor data and a larger assortment of body sounds relative to the scenario.

Future research could also be conducted to use this simulation approach to develop emotionally engaging scenarios which may prepare students to better cope with a high emotion real-life situation. (Ignacio et al., 2015).

## Data Availability

The datasets used and/or analysed during the current study are available from the corresponding author on reasonable request.
